# Provisional Use of CLSI-Approved Quality Control Strains for Antimicrobial Susceptibility Testing of *Mycoplasma* (*‘Mesomycoplasma’*) *hyorhinis*

**DOI:** 10.3390/microorganisms9091829

**Published:** 2021-08-28

**Authors:** Lisa Käbisch, Anne-Kathrin Schink, Corinna Kehrenberg, Stefan Schwarz

**Affiliations:** 1 Department of Veterinary Medicine, Institute of Microbiology and Epizootics, Freie Universität Berlin, 14163 Berlin, Germany; lisa.kaebisch@fu-berlin.de (L.K.); stefan.schwarz@fu-berlin.de (S.S.); 2 Department of Veterinary Medicine, Institute for Veterinary Food Science, Justus-Liebig-University Gießen, 35392 Gießen, Germany; corinna.kehrenberg@vetmed.uni-giessen.de

**Keywords:** antimicrobial susceptibility testing (AST), broth microdilution, *Mycoplasma hyorhinis*, quality control strains

## Abstract

Antimicrobial susceptibility testing (AST) should be conducted in a standardized manner prior to the start of an antimicrobial treatment. For fastidious bacteria, such as porcine *Mycoplasma* (*‘Mesomycoplasma’*) spp., specifically *M. hyorhinis*, neither guidelines or standards for the performance of AST, nor quality control strains for the validation of AST results are approved by organizations like the Clinical and Laboratory Standards Institute (CLSI) or the European Committee on Antimicrobial Susceptibility Testing (EUCAST). The CLSI- and EUCAST-approved quality control strains *Enterococcus faecalis* ATCC 29212 and *Staphylococcus aureus* ATCC 29213 were chosen to validate AST by broth microdilution using modified Friis broth, developed as growth medium for porcine *Mycoplasma* (*‘Mesomycoplasma’*) spp. The antimicrobial agents doxycycline, enrofloxacin, erythromycin, florfenicol, gentamicin, marbofloxacin, tetracycline, tiamulin, tilmicosin, tulathromycin, and tylosin were examined using customized Sensititre^TM^ microtiter plates. Minimal inhibitory concentrations, determined after 24, 48, and 72 h, were mostly within the CLSI-approved quality control ranges for defined antimicrobial agents. We propose the use of the combination of *E. faecalis* ATCC 29212 and *S. aureus* ATCC 29213 as surrogate quality control strains for the validation of future AST results obtained for *M. hyorhinis* by broth microdilution using modified Friis broth.

## 1. Introduction

*Mycoplasma* (*‘Mesomycoplasma’*) *hyorhinis*, first described by Switzer in 1955, is a widely distributed pathogen in pigs [1,2]. It is associated with respiratory diseases, arthritis, and polyserositis, as well as systemic infections in piglets, thereby decreasing animal welfare and causing economic losses [3]. *M. hyorhinis* is closely related to *M. hyopneumoniae*, another pathogenic *Mycoplasma* spp. in pigs, associated with enzootic pneumonia (EP) and the porcine respiratory disease complex (PRDC). Together with other *Mycoplasma* spp., they belong to the class of Mollicutes. Their main characteristics are a very small size (0.2–0.3 µm), a small genome (580,000–1,350,000 bp), as well as their lack of a cell wall. Gupta et al. assigned *M. hyorhinis* and *M. hyopneumoniae* to the novel genus *Mesomycoplasma.* The reclassification is still under debate [4,5,6].

For a rational use of antimicrobial agents in food-producing animals, bacterial pathogens should be isolated, identified, and their antimicrobial susceptibility determined. Organizations like the Clinical and Laboratory Standards Institute (CLSI) and the European Committee on Antimicrobial Susceptibility Testing (EUCAST) provide antimicrobial susceptibility testing (AST) standards for many bacterial pathogens. Publications presenting AST data of *M. hyorhinis* are available, though standardized methods are not yet established. In contrast, various methods have been used, which renders the comparison of the corresponding results impossible. Next to the AST procedure, approved standards commonly provide information on quality control (QC) strains, which are used as an internal control to validate the test system. However, no QC strains have been approved for *M. hyorhinis* or other porcine *Mycoplasma* (*‘Mesomycoplasma’*) spp.

The Friis broth, specifically developed for porcine *Mycoplasma* (*‘Mesomycoplasma’*) spp., is especially suited for culturing these fastidious organisms. To our knowledge, this complex broth medium best provides the needed nutrients for porcine *Mycoplasma* (*‘Mesomycoplasma’*) spp. [7]. The modified Friis broth used in this study, based on the recommendation of the German Collection of Microorganisms and Cell Cultures GmbH (DSMZ, Braunschweig, Germany) for culturing porcine *Mycoplasma* (*‘Mesomycoplasma’*) spp. is—compared to the original Friis broth—free of antimicrobial agents and consequently better suited for AST [8].

In this study, we introduce a first step towards a standardized AST procedure for *M. hyorhinis* using a broth microdilution method with the modified Friis broth as test medium. The widely used QC strains *Enterococcus faecalis* ATCC 29212 and *Staphylococcus aureus* ATCC 29213 are approved by CLSI and EUCAST, alongside available QC ranges in cation-adjusted Mueller-Hinton broth. We tested these two QC strains for their performance in modified Friis medium and propose the provisional combinational use of these two QC strains as surrogate quality controls until a *M. hyorhinis* QC strain is approved.

## 2. Materials and Methods

### 2.1. Bacterial Strains

The QC strains *Enterococcus faecalis* ATCC 29212 (DSM 2570) and *Staphylococcus aureus* ATCC 29213 (DSM 2569) were purchased from the DSMZ, Braunschweig, Germany.

### 2.2. Media

The modified Friis broth, based on the formula initially provided by the DSMZ, facilitates the growth of porcine *Mycoplasma* (*‘Mesomycoplasma’*) spp. and is used as test medium [7,8]. For approximately 176 mL of modified Friis broth, the broth base is comprised of 0.82 g of porcine brain heart infusion (Sigma-Aldrich Chemie GmbH, Taufkirchen, Germany), 0.87 g of Difco^TM^ Mycoplasma PPLO Broth w/o CV (BD, NJ, USA), 50 mL of Hank’s Balanced Salt Solution (Gibco^®^, Thermo Fisher Scientific, Waltham, MA, USA), and 78 mL of deionized water. The pH was set to 7.4. After autoclaving (15 min at 121 °C, 1 bar) and cooling, the following supplements were added: 40.6 mL of heat inactivated porcine serum (Gibco^®^), 4.49 mL of 25% autoclaved yeast extract solution (Carl Roth GmbH + Co. KG, Karlsruhe, Germany), 0.36 mL of 1% filter sterilized phenol red solution (phenol red sodium salt, Carl Roth GmbH; 22 µm ROTILABO^®^ MCE syringe filter, Carl Roth GmbH), and 0.284 mL of deionized water. For colony counts, Columbia blood agar (Oxoid^TM^, Wesel, Germany) was used.

The porcine serum was heat inactivated by incubating the serum in a 56 °C water bath for 30 min. To deprive the sera of the lipid aggregates, the heat inactivated sera were filtered (70 µm cell strainer, Sarstedt, Nümbrecht, Germany) and centrifuged at 400× *g* for 2 min. The clear filtrates were aliquoted and stored until use at −20 °C.

In comparison to the DSMZ recipe, we increased the amount of porcine serum (from 18.35% to 23.13%), yeast extract (from 2.03% to 2.56%), and doubled the amount of phenol red solution. A serum concentration above 20% allows the storage of the bacterial cultures at −80 °C without additives and the increased amount of phenol red solution facilitates a more convenient readout of the microtiter plates.

### 2.3. Antimicrobial Susceptibility Testing

The AST of *E. faecalis* ATCC 29212 and *S. aureus* ATCC 29213 was conducted according to CLSI standards [9]. The inoculum was prepared using a fresh overnight culture of the respective strain. Single colony material was inoculated into 0.85% NaCl solution and adjusted photometrically (V-1200 Spectrophotometer, VWR, Leuven, Belgium) to an optical density between 0.08 and 0.13 at 625 nm, corresponding to 0.5 McFarland standard. The suspension was used to prepare the inoculum by pipetting 5 µL of suspension per mL modified Friis broth, resulting in a final concentration of approximately 5 × 10^5^ colony forming units (cfu)/mL. The AST was performed using customized microtiter plates (Sensititre^TM^, Thermo Fisher Scientific) [10]. They were inoculated with 50 µL of inoculum suspension per well, sealed with an adhesive foil, and incubated at 35 ± 2 °C for 18, 48, and 72 h, respectively. The extended incubation time of 48 and 72 h was chosen due to the prolonged incubation time required for the growth of *M. hyorhinis.* For quality control purposes, the colony count was assessed as described below.

The following antimicrobial agents were tested: clindamycin (0.03–64 mg/L), doxycycline (0.06–128 mg/L), enrofloxacin (0.008–16 mg/L), erythromycin (0.015–32 mg/L), florfenicol (0.12–256 mg/L), gentamicin (0.12–256 mg/L), marbofloxacin (0.008–16 mg/L), tetracycline (0.12–256 mg/L), tiamulin (0.03–64 mg/L), tilmicosin (0.06–128 mg/L), tulathromycin (0.06–32 mg/L), and tylosin (0.06–128 mg/L). Minimal inhibitory concentrations (MICs) for *E. faecalis* ATCC 29212 and *S. aureus* ATCC 29213 were determined according to CLSI documents [9].

### 2.4. Colony Counts

To verify the correct inoculum size, cfu counts of *E. faecalis* ATCC 29212 and *S. aureus* ATCC 29213 were determined according to CLSI standards [9]. In total, 10 µL of the inoculum suspension were diluted in 10 mL of 0.85% NaCl solution, followed by plating 100 µL of this solution onto Columbia blood agar (Oxoid^TM^). The plates were incubated at 35 ± 2 °C for 18 h. A count of 20–80 colonies was acceptable.

## 3. Results and Discussion

The antimicrobial agents investigated were chosen to represent the antimicrobial agents of interest for future testing of the *M. hyorhinis* ATCC 17981 type strain, as well as a variety of field isolates of different geographic origins. Tetracyclines, macrolides, and pleuromutilins were of particular interest, since antimicrobial agents of these classes are commonly used to treat porcine respiratory infections caused by *Mycoplasma* (*‘Mesomycoplasma’*) spp. Erythromycin was included because several studies reported an intrinsic resistance to this 14-membered macrolide in *M. hyopneumoniae* as well as *M. hyorhinis* [11,12,13,14]. Lincosamides are also of interest for the treatment of porcine *Mycoplasma* (*‘Mesomycoplasma’*) spp. Though not approved for use in food-producing animals, clindamycin was tested as the class representative of the lincosamides [15].

Fourteen independent tests of each QC strain were conducted in modified Friis broth. After 18 h of incubation, the following strain-antimicrobial agent combinations were mostly within CLSI-defined quality control ranges: *E. faecalis* ATCC 29212—gentamicin, erythromycin, tilmicosin, tulathromycin, tylosin, florfenicol, enrofloxacin, doxycycline (Table 1) and *S. aureus* ATCC 29213—clindamycin, tiamulin, marbofloxacin, tetracycline (Table 2). In two of these tests, the MIC values of *E. faecalis* ATCC 29212 for tilmicosin were one dilution step below the given QC range after 18 h of incubation. For *S. aureus* ATCC 29213, none of the tested antimicrobial agents deviated from the given QC ranges. With the increasing incubation times, a slight general increase of MIC values for both strains and most antimicrobial agents was observed (Table 1 and Table 2). In these cases, we evaluated whether the respective strain-antimicrobial agent combination was (i) still within the respective QC range and (ii) whether the individual increase represented a minor (1 dilution step) or a major (≥2 dilution steps) change.

For *E. faecalis* ATCC 29212, three enrofloxacin MIC values read after 72 h of incubation were increased by one dilution step above the approved QC range. For all other strain-antimicrobial agent combinations, the MIC values were stable or increased by one dilution step at most, still within the QC ranges approved by CLSI (Table 1) [15].

For *S. aureus* ATCC 29213, an increase of the MIC values by one dilution step above the given QC range was observed for the following incubation time-antimicrobial agent combinations: marbofloxacin after 48 h (one test), and clindamycin and tetracycline after 72 h (one test each).

The results of the QC strains *E. faecalis* ATCC 29212 and *S. aureus* ATCC 29213 obtained with the modified Friis broth were repeatable. With very few exceptions (two test results for tilmicosin and *E. faecalis* ATCC 29212), the obtained MIC values of the surrogate QC strains in modified Friis broth read after 18 h of incubation were within the CLSI-approved QC ranges of incubation for 18 h in cation-adjusted Mueller Hinton broth. Since staphylococci and enterococci do not need incubation periods longer than 24 h at maximum, no information about the potential change of the MIC values of the two QC strains after incubation for 48 or 72 h in cation-adjusted Mueller Hinton broth are available. After prolonged incubation times of 48 and 72 h in modified Friis broth, most MIC values were still within the CLSI-approved QC ranges. Time-dependent increases in the MIC values of both QC strains represented minor changes of only one dilution step above the CLSI-approved QC ranges (Table 1 and Table 2).

For *E. faecalis* ATCC 29212 and *S. aureus* ATCC 29213, the modified Friis broth was suitable for AST by broth microdilution in this study. Though being a very complex medium, compared to commercial ready-to-use media facilitating the growth of porcine *Mycoplasma* (*‘Mesomycoplasma’*) spp., the formula is available and thereby controllable, while ready-to-use media do not always provide a detailed description of their ingredients. The modified Friis broth is also free of antimicrobial agents, usually added to complex *Mycoplasma* (*‘Mesomycoplasma’*) spp.-specific broth media. The addition of antimicrobial agents renders the broth medium unsuitable for AST applications. According to the recipe provided by Niels Friis, bacitracin and methicillin were added to support the selective growth of porcine *Mycoplasma* (*‘Mesomycoplasma’*) spp., thereby interfering with the cell wall synthesis of contaminants [7]. *Mycoplasma* (*‘Mesomycoplasma’*) spp. are intrinsically resistant to antimicrobial agents targeting the cell wall synthesis. The QC strains *E. faecalis* ATCC 29212 and *S. aureus* ATCC 29213, on the contrary, are susceptible to cell wall-active antimicrobial agents and thus would be inhibited by the addition of bacitracin, methicillin, or related antimicrobial agents.

In the past, several published studies presented antimicrobial susceptibility test results of *M. hyorhinis* [12,13,16,17,18,19,20]. Unfortunately, the results of most of these studies are not comparable, due to differing parameters like test media, inoculum size or preparation, incubation temperatures, or incubation times. Moreover, some studies lack QC strains in general or they used different *M. hyorhinis* strains, which displayed different MIC values.

No approved QC strain for porcine *Mycoplasma* (*‘Mesomycoplasma’*) spp. is yet available. The first steps towards establishing a standardized broth microdilution method for AST would be to determine the suitability of the modified Friis broth for *M. hyorhinis* type and field strains as well as the definition of a QC strain and accompanying QC ranges. For proper control of these initial efforts, we chose to control and validate our tests by using surrogate QC strains. The combination of CLSI-approved QC strains *E. faecalis* ATCC 29212 and *S. aureus* ATCC 29213 best mirrored the antimicrobial agent profile of interest for future *M. hyorhinis* AST. Therefore, we suggest using the proposed antimicrobial agent-QC strain combinations, as shown in Table 1 and Table 2, in ASTs of *M. hyorhinis*.

In conclusion, the results of this study showed that the two widely used QC strains *E. faecalis* ATCC 29212 and *S. aureus* ATCC 29213 produced MIC values in modified Friis broth that were largely within the CLSI-approved QC ranges for MIC values in cation-adjusted Mueller Hinton broth. Hence, they might be used for QC in AST of *M. hyorhinis* isolates until more suitable species-specific QC strains are identified and approved.

## Figures and Tables

**Table 1:** Distribution of MIC values for *E. faecalis* ATCC 29212 read after 18, 48, and 72 h, respectively. Green shading and vertical bars indicate the CLSI-approved QC ranges for 18 h of incubation. The approved QC ranges were also applied to the time points 48 and 72 h. Fourteen independent test results are shown.

Number of Tests and MIC Values Obtained (mg/L)																		
Antimicrobial Agent	Incubation Time	0.008	0.015	0.03	0.06	0.12	0.25	0.5	1	2	4	8	16	32	64	128	256	512
**Aminoglycosides**																		
Gentamicin	18 h					-	-	-	-	-	-	-	14	-	-	-	-	-
	48 h					-	-	-	-	-	-	-	14	-	-	-	-	-
	72 h					-	-	-	-	-	-	-	14	-	-	-	-	-
**Macrolides**																		
Erythromycin	18 h		-	-	-	-	-	-	-	14	-	-	-	-	-			
	48 h		-	-	-	-	-	-	-	7	7	-	-	-	-			
	72 h		-	-	-	-	-	-	-	5	9	-	-	-	-			
Tilmicosin	18 h				-	-	-	-	-	-	2	12	-	-	-	-	-	
	48 h				-	-	-	-	-	-	-	8	6	-	-	-	-	
	72 h				-	-	-	-	-	-	-	1	13	-	-	-	-	
Tulathromycin	18 h				-	-	-	-	-	-	12	2	-	-	-			
	48 h				-	-	-	-	-	-	9	5	-	-	-			
	72 h				-	-	-	-	-	-	8	6	-	-	-			
Tylosin	18 h				-	-	-	-	13	1	-	-	-	-	-	-	-	
	48 h				-	-	-	-	6	8	-	-	-	-	-	-	-	
	72 h				-	-	-	-	2	12	-	-	-	-	-	-	-	
**Phenicols**																		
Florfenicol	18 h					-	-	-	-	-	14	-	-	-	-	-	-	-
	48 h					-	-	-	-	-	14	-	-	-	-	-	-	-
	72 h					-	-	-	-	-	12	2	-	-	-	-	-	-
**Fluoroquinolones**																		
Enrofloxacin	18 h	-	-	-	-	-	2	12	-	-	-	-	-	-				
	48 h	-	-	-	-	-	-	7	7	-	-	-	-	-				
	72 h	-	-	-	-	-	-	1	10	3	-	-	-	-				
**Tetracyclines**																		
Doxycycline	18 h				-	-	-	-	-	-	-	10	4	-	-	-	-	
	48 h				-	-	-	-	-	-	-	-	12	2	-	-	-	
	72 h				-	-	-	-	-	-	-	-	9	5	-	-	-	

**Table 2:** Distribution of MIC values for *S. aureus* ATCC 29213 read after 18, 48, and 72 h, respectively. Green shading and vertical bars indicate CLSI-approved QC ranges for 18 h of incubation. The approved QC ranges were also applied to the time points 48 and 72 h. Fourteen independent test results are shown.

Number of Tests and MIC Values Obtained (mg/L)																		
Antimicrobial Agent	Incubation Time	0.008	0.015	0.03	0.06	0.12	0.25	0.5	1	2	4	8	16	32	64	128	256	512
**Lincosamides**																		
Clindamycin	18 h			-	-	10	4	-	-	-	-	-	-	-	-	-		
	48 h			-	-	1	13	-	-	-	-	-	-	-	-	-		
	72 h			-	-	-	13	1	-	-	-	-	-	-	-	-		
**Pleuromutilins**																		
Tiamulin	18 h			-	-	-	-	13	1	-	-	-	-	-	-	-		
	48 h			-	-	-	-	-	14	-	-	-	-	-	-	-		
	72 h			-	-	-	-	-	11	3	-	-	-	-	-	-		
**Fluoroquinolones**																		
Marbofloxacin	18 h	-	-	-	-	-	-	14	-	-	-	-	-	-				
	48 h	-	-	-	-	-	-	13	1	-	-	-	-	-				
	72 h	-	-	-	-	-	-	13	1	-	-	-	-	-				
**Tetracyclines**																		
Tetracycline	18 h					-	-	14	-	-	-	-	-	-	-	-	-	-
	48 h					-	-	1	13	-	-	-	-	-	-	-	-	-
	72 h					-	-	-	13	1	-	-	-	-	-	-	-	-

## Data Availability

All data are contained within the article.
